# Politicians’ views on societal responsibility and possibility to promote newly arrived migrants’ health in Sweden

**DOI:** 10.1093/heapro/daab199

**Published:** 2021-12-13

**Authors:** Sara Svanholm, Heidi Carlerby, Eija Viitasara

**Affiliations:** Department of Health Sciences, Mid Sweden University, Homgatan 10, 851 70 Sweden

**Keywords:** politicians, migrants, cluster analysis, health promotion, integration

## Abstract

Newly arrived migrants in Sweden risk facing ill health. Politicians at the local and regional levels are involved in many decisions regarding the social determinants of health. The aim of this study was to explore politicians’ views on different societal actors’ responsibility and possibility to promote newly arrived migrants’ health. Data were collected through online questionnaires completed by 667 politicians from municipality and regional councils in northern Sweden. Bivariate analysis was performed using the Wilcoxon signed-rank test. Multivariate analyses were performed using cluster analysis and binary logistic regression analysis. The results show that politicians generally rate societal actors’ responsibility and possibility to promote the general population’s health higher than newly arrived migrants’ health. Moreover, they consider societal actors’ responsibility to be greater than their possibility to promote health. Factors significantly contributing to politicians’ high ratings of societal responsibility and possibility are attitude (odds ratio [OR] = 2.156, 95% confidence interval [CI]: 1.306–3.558), specific knowledge of newly arrived migrants’ health status (OR = 1.528, 95% CI: 1.005–2.323), personal interest in public health (OR = 2.452, 95% CI: 1.460–4.119), being a municipality politician (OR = 1.659, 95% CI: 1.031–2.670) and being female (OR = 1.934, 95% CI: 1.333–2.806). This study shows that politicians generally rate societal responsibility and possibility to promote newly arrived migrants’ health rather high. Personal characteristics are important for politicians’ high or low ratings of responsibility and possibility, suggesting insufficient structural support for politicians in health promotion.

## INTRODUCTION

Health inequities, or systematic, socially produced and unfair inequalities in health (Dahlgren and Whitehead, 2007), are a problem both globally and in Sweden (Swedish Commission for Equity in Health, 2017). Migrants face inequities both in social determinants, such as unemployment (Rechel *et al.*, 2013), housing situation, language skills, social support and discrimination (Hynie, 2018), and in direct health outcomes (Rechel *et al.*, 2013). The risk of ill health increases with the duration of stay in a new country (Norredam *et al.*, 2014). In Sweden, non-Western migrants have an increased risk of ill health compared to Swedish natives (Helgesson *et al.*, 2019).

Responsibility is a concept that has been theorized and discussed with regard to health. With a growing understanding of the importance of social determinants for public health, the view of who is responsible for health has shifted and widened. Studies have shown that emphasizing individuals’ role and responsibility in health can increase empowerment and the feeling of being in control of one’s own health and destiny. However, research has also shown that since societal structures heavily influence the population’s health, the individual cannot have a central role in public health (Wikler, 2002). Health-promoting initiatives solely built on promoting individuals’ own responsibility, especially moral responsibility, for their health, have been deemed ineffective (Brown *et al.*, 2019).

A category of actors that can affect many social determinants of health in the Swedish context is that of politicians at the municipality and regional levels. Municipalities are responsible for determinants such as school and education, housing, city planning and elderly care (Swedish Association of Local Authorities and Regions, 2021a), while regions are responsible for healthcare and regional development (Swedish Association of Local Authorities and Regions, 2021b).

A country’s integration policies affect both self-reported and objective health measures for migrants in Europe (Giannoni *et al.*, 2016). Restrictive policies regarding welfare and other non-health-targeted policies are associated with poorer health outcomes for migrants (Juárez *et al.*, 2019). Integration processes are thus important in combating the health inequities facing migrants (Giannoni *et al.*, 2016).

In the integration process in Sweden, municipalities are among the major actors, while the regions and the healthcare sector are involved on a case-by-case basis (Government Office of Sweden, 2010). Persons who have arrived as asylum seekers and relatives of previous asylum seekers are called newly arrived migrants for 2 years after receiving a residence permit (Government Office of Sweden, 2017). During this time, they are eligible to participate in a civic orientation program (the Establishment Program) to enhance their employability, learn Swedish and familiarize themselves with Swedish society (Public Employment Services, 2021).

Since ill health increases with the time spent in the country (Norredam *et al.*, 2014), the establishment time is important for reaching newly arrived migrants with targeted programs. Research has shown that municipality and regional politicians consider newly arrived migrants’ health only partly in their decision-making (Svanholm *et al.*, 2021). However, their views on their own and other societal actors’ responsibility and possibility to promote health have been less researched.

## AIM

The aim of this study was to explore politicians’ views on various societal actors’ responsibility and possibility to promote newly arrived migrants’ health. The specific research questions were as follows:

- Do politicians rate societal actors’ responsibility and possibility to promote newly arrived migrants’ health differently than they rate the same actors’ responsibility and possibility to promote the population’s health?
- Is there a significant difference between how politicians rate societal actors’ responsibility versus possibility to promote newly arrived migrants’ health?
- What factors affect how politicians rate societal actors’ responsibility and possibility to promote newly arrived migrants’ health?

## METHOD

### Data collection

Data were collected through an electronic questionnaire during 2019. The questionnaire was developed following interviews with politicians and focused on politicians’ views on their role, responsibility and possibility to promote newly arrived migrants’ health. The development of the questionnaire has been described previously (Svanholm *et al.*, 2021). The questionnaire consisted of 49 items divided into five parts: politicians’ views on health and health promotion, affecting health as a politician, collaboration between actors, the politics of health, and municipality and regional organization and its prerequisites. The items were mainly in the form of statements rated on Likert scales on which the participants rated their degree of agreement. There were also three open-ended questions, as well as room for the participants to clarify their answers with written text. Several questions and statements were doubled, allowing the participants to rate items for both newly arrived migrants and the general population.

The questionnaire was e-mailed to all politicians (2744 in total) in the municipality and regional councils in the four northernmost counties of Sweden. In cases in which a politician held seats in both a municipality and a regional council, they were asked to choose one and respond to the questionnaire from that perspective. To ensure a sufficient sample, both original members and alternates were included. The contact information of each politician was obtained from the respective municipality or region.

The questionnaire was open for 6 weeks between April and May 2019. Three reminders were sent during that period. A total of 667 politicians (24.3% response rate) returned completed questionnaires and were included in the study.

### Participants

Of the participants, 83.3% held seats in municipality councils, 46.3% were women and 55.2% were aged 55 years or older. In these respects, there were no differences between the sample and the general population (Svanholm *et al.*, 2021). Approximately 30% of the participants were on their first term and had no previous experience in councils, while 36% had served three or more terms.

In rating their experience of working on public health, 27.5% of the participants reported that they had worked on public health issues as politicians, 12.8% in another capacity and 11.3% as both politicians and in another capacity, while 40.5% had no previous experience. Among the participants, 80.1% reported that they had a personal interest in public health. Regarding integration, 27.5% of the participants reported having worked on integration issues as politicians, 24.4% in another capacity and 10.2% both as politicians and in another capacity, while 37.9% had no previous experience. Approximately 81% reported having a personal interest in integration.

### Data analysis

The data were transferred from Netigate to IBM SPSS Statistics 27. The variables used are shown in Table 1. Univariate data were presented as frequencies and percentages. Bivariate analysis was performed using the Wilcoxon signed-rank test. The significance level was set to *P* < 0.05. Cohen’s coefficient was used to assess the effect size [(Cohen, 1988, cited in Pallant, 2016), pp. 234–236].

**Table 1:** Variables included in the study

Variable	Questionnaire item	Coded	Dichotomized code
Responsibility—population	How large responsibility do you consider the following societal actors having for the population’s health?^c^	1–5 Likert scale^a^	N/A
Responsibility—newly arrived migrants	How large responsibility do you consider the following societal actors having for newly arrived migrants’ health?^c^	1–5 Likert scale^a^	N/A
Possibility—population	How large possibility do you consider the following societal actors to have to promote population’ health?^c^	1–5 Likert scale^a^	N/A
Possibility—newly arrived migrants	How large possibility do you consider the following societal actors to have to promote newly arrived migrants’ health? ^c^	1–5 Likert scale^a^	N/A
Attitude	The effects on newly arrived migrants’ health are important for my political decision-making.	1–5 Likert scale^a^	0 = 1–3, not important; 1 = 4–5, important
Knowledge of newly arrived migrants’ health	I consider myself to have a clear picture of newly arrived migrants’ health status in the municipality/region where I am a politician.	1–5 Likert scale^a^	0 = 3–5, no knowledge; 1 = 1–2, knowledge
General health knowledge index	Index^d^	Range: 1–18	N/A
Experience in public health	Do you have previous experience working on public health issues?	1 = yes, as a politician; 2 = yes, in another capacity; 3 = no	0 = 3, no experience; 1 = 1–2, experience
Experience in integration issues	Do you have previous experience working on integration issues?	1 = yes, as a politician; 2 = yes, in another capacity; 3 = no	0 = 3, no experience; 1 = 1–2, experience
Experience in politics	What is your previous experience as a politician in the municipality/regional council?	1 = none, first term; 2 = one term; 3 = two terms; 4 = three or more terms	0 = 1–2, little experience; 1 = 3–4, considerable experience
Interest in public health	I am personally interested in public health as a political issue.	1–5 Likert scale^b^	0 = 1–3, not interested; 1 = 4–5, interested
Interest in integration	I am personally interested in integration as a political issue.	1–5 Likert scale^b^	0 = 1–3, not interested; 1 = 4–5, interested
Council membership	What is your current political role/assignment?	1 = ordinary member of municipality council; 2 = alternate municipality council; 3 = ordinary member of regional council; 4 = alternate regional council	0 = 3–4, municipality politician; 1 = 1–2, regional politician
Gender	What gender do you identify with?	1 = female; 2 = male; 3 = other	0 = 2, male; 1 = 1, female
Age	Age?	1 = 18–24; 2 = 25–34; 3 = 35–44; 4 = 45–54; 5 = 55–64; 6 = 65–74; 7 =≥75	0 = 1–4, <55 years; 1 = 5–7, ≥55 years

a 1= no responsibility/possibility; 2 = small responsibility/possibility; 3 = some responsibility/possibility; 4 = rather great responsibility/possibility; 5 = great responsibility/possibility.

b 1 = fully agree; 2 = partly agree; 3 = neither agree nor disagree; 4 = partly disagree; 5 = fully disagree.

c The politicians were asked to rate the following actors: individuals, municipalities, municipality politicians, regions, regional politicians, the state, civil society and private actors.

d Index based on the following items: ‘I consider myself to have enough knowledge to judge whether a political decision that I make affects the population’s health’; ‘I consider myself to have enough knowledge to judge how a political decision that I make affects the population’s health’; ‘I understand the differences between health promotion and disease prevention’, ‘I understand the municipality’s/region’s responsibility for the population’s health according to the new national public health political goals established in 2018’; ‘I consider myself to have a clear picture of the health status of the general population in the municipality/region where I am a politician’ (Svanholm *et al.*, 2021).

A two-phase cluster analysis was performed to derive cluster profiles of the rated responsibility and possibility to promote newly arrived migrants’ health. All included variables were of a similar type and ratio and did not need to be standardized before the analysis. A hierarchical cluster analysis using Ward’s method for clustering was performed to identify the number of clusters in the data. Then, a *k*-means (nonhierarchical) cluster analysis was performed using the previously identified number of clusters (two clusters) [(Henry *et al.*, 2005; Hair *et al.*, 2010), pp. 415–474]. Each participant’s cluster profile was saved.

The validity of the cluster solution was assessed through cross-validation, first by performing the cluster analysis using a random sample of half of the participants. The rest of the participants were then used in a second analysis, and the results were compared. No significant differences were observed [(Henry *et al.*, 2005; Hair *et al.*, 2010), pp. 449–450]. All participants were included in the analysis.

A logistic regression analysis with the two clusters as outcome variables was then performed to examine which variables contributed to politicians’ high ratings of societal responsibility and possibility to promote health. The analysis was performed in one step, and adjusted odds ratios (OR) and 95% confidence intervals (CI) were calculated. For the degree of model explanation, Cox and Snell’s *R*^2^ and Nagelkerke’s *R*^2^ were calculated. For model significance, omnibus tests of model coefficients were used, and goodness of fit was evaluated using the Hosmer–Lemeshow test [(Hosmer *et al.*, 2013; Pallant, 2016), pp. 169–181].

### Ethics

Ethics approval for the study was obtained from the Research Ethics Committee of Mid Sweden University (MIUN 2019/6). The study was designed in accordance with the ethical standards of the Swedish National Research Committee (Swedish Research Council, 2017) and the 1964 Helsinki Declaration and its later amendments (World Medical Association, 2018). All participants were informed in writing about the aim of the study and the intent to publish the results and were assured that participation was voluntary and confidential. The participants consented to participate by submitting the completed questionnaire.

## RESULTS

Politicians generally rated most social actors’ responsibility for health higher for the general population than for newly arrived migrants. The largest effect size in difference was in individuals’ responsibility for health. The participants believed that individuals in the general population had greater responsibility than individuals among newly arrived migrants. However, the effect size was relatively small (0.25). There were also significant differences between the rated responsibility of regions, regional politicians, civil society, and private actors (Table 2).

**Table 2:** Politicians’ rated responsibility for the population’s versus newly arrived migrants’ health

	Population	Newly arrived migrants	*Z*	*P*	*N*	*R*a
Individuals	5	4	−8.967	0.001	655	0.248
Municipalities	4	4	−1.108	0.268	657	0.031
Municipality politicians	4	4	−1.489	0.137	656	0.041
Regions	5	5	−6.317	0.001	654	0.175
Regional politicians	5	4	−5.456	0.001	656	0.151
State	5	5	−1.276	0.202	656	0.035
Civil society	4	3	−2.154	0.031	655	0.060
Private actors	3	3	−6.971	0.001	655	0.193

a Cohen’s coefficient. Small, *r* = 0.10–0.29; medium, *r* = 0.30–0.49; large, *r* = 0.50–1.0.

Politicians’ views on societal possibility to promote health showed similarities with their views on responsibility. They generally rated the possibility to promote the general population’s health higher than newly arrived migrants’ health. Individuals, the municipalities, regions and regional politicians, civil society and private actors were rated significantly higher for the population than for newly arrived migrants. However, the effect sizes were relatively small (Table 3).

**Table 3:** Politicians’ rated possibility to promote the population’s versus newly arrived migrants’ health

	Population	Newly arrived migrants	*Z*	*P*	*N*	*R*a
Individuals	4	4	−5.227	0.001	651	0.145
Municipalities	4	4	−1.983	0.047	655	0.055
Municipality politicians	4	4	−0.610	0.542	653	0.017
Regions	4	4	−4.800	0.001	655	0.133
Regional politicians	4	4	−3.460	0.001	654	0.096
State	5	5	−0.835	0.404	655	0.023
Civil society	4	3	−2.098	0.036	654	0.058
Private actors	3	3	−3.892	0.001	653	0.108

a Cohen’s coefficient. Small, *r* = 0.10–0.29; medium, *r* = 0.30–0.49; large, *r* = 0.50–1.0.

The politicians rated societal responsibility higher than societal possibility to promote newly arrived migrants’ health. The difference was significant for all actors except for civil society and private actors. However, the effect sizes were relatively small, but larger than the previously reported effect sizes (Table 4).

**Table 4:** Politicians’ rated societal responsibility versus possibility to promote newly arrived migrants’ health

	Responsibility	Possibility	*Z*	*P*	*N*	*R*a
Individuals	4	4	−8.698	0.001	652	0.241
Municipalities	4	4	−8.558	0.001	654	0.237
Municipality politicians	4	4	−9.657	0.001	653	0.267
Regions	5	4	−8.307	0.001	655	0.230
Regional politicians	4	4	−9.945	0.001	653	0.275
State	5	5	−6.091	0.001	653	0.169
Civil society	3	3	−0.221	0.825	653	0.006
Private actors	3	3	−1.340	0.180	653	0.037

a Cohen’s coefficient. Small, *r* = 0.10–0.29; medium, *r* = 0.30–0.49; large, *r* = 0.50–1.0.

**Fig. 1:**
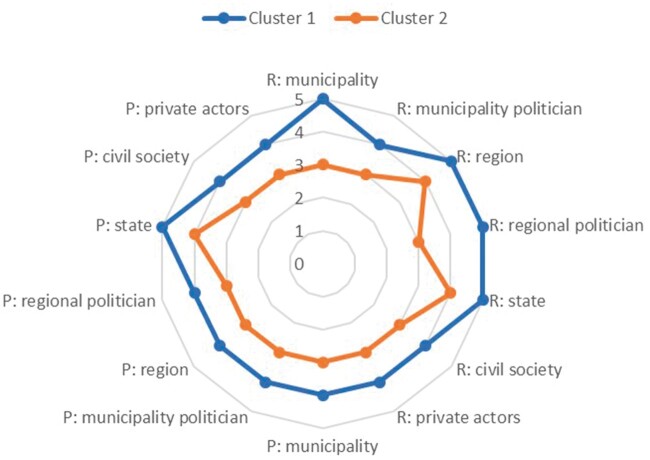
Cluster analysis results. Median of each cluster regarding how the politicians rated each actor’s responsibility (R) and possibility (P) to promote newly arrived migrants’ health. The differences in the medians of all variables between the two clusters were statistically significant.

Two clusters were identified in the data (Figure 1). One cluster consisted of 401 politicians who rated societal responsibility and possibility high on all included variables. The other cluster included 243 politicians who rated societal responsibility and possibility low.

The most important factors determining whether politicians belonged to the cluster that rated the responsibility and possibility to promote newly arrived migrants’ health high were attitude, specific knowledge of newly arrived migrants’ health status, personal interest in public health, being a municipality politician and being female (Table 5).

**Table 5:** Odds of politicians belonging to the cluster that rated societal responsibility and ability to promote newly arrived migrants’ health high

		95% CI for Exp(*B*)	
	OR	Lower	Upper
Attitude	2.156	1.306	3.558
Knowledge of newly arrived migrants’ health	1.528	1.005	2.323
General health knowledge	0.974	0.899	1.056
Previous experience in public health	1.113	0.718	1.727
Previous experience in integration	0.870	0.563	1.347
Personal interest in public health	2.452	1.460	4.119
Personal interest in integration	1.639	0.954	2.814
Type of politician	1.659	1.031	2.670
Tenure as a politician	0.801	0.539	1.191
Gender	1.934	1.333	2.806
Age	1.020	0.689	1.509
Constant	0.193		

Omnibus test of model coefficients model *χ*^2^ = 89.554 (degrees of freedom [df] = 11, *P* < 0.001). Cox and Snell’s *R*^2^ = 0.139; Nagelkerke’s *R*^2^ = 0.190; Hosmer–Lemeshow *χ*^2^ = 8.004 (df = 8, *P* = 0.433). Correct classifications: 70.7%.

## DISCUSSION

This study shows that politicians generally rate societal actors’ responsibility in health promotion rather high. Moreover, the results indicate that politicians rate the responsibility and possibility to promote the general population’s health higher than newly arrived migrants’ health. The state is the actor with the highest rated responsibility for both the general population’s and newly arrived migrants’ health. Generally, politicians consider that actors have greater responsibility than possibility to promote newly arrived migrants’ health. Civil society and private actors are rated lower but equal in responsibility and possibility. Factors important for politicians’ high ratings of societal responsibility and possibility are attitude, specific knowledge of newly arrived migrants’ health, personal interest in public health, being a politician in a municipality council and being female.

Previous research and ethical discussions have often focused on access to healthcare with regard to social responsibility for health (Resnik, 2007). In this study, politicians rated the regions, which are responsible for health care in Sweden, as having both great responsibility and great possibility to promote health. However, other actors at both the national and local levels were also rated as closely involved. The low responsibility and possibility ratings for private actors are in line with previous research highlighting issues related to involving private actors in public health actions, especially when the actions go against the actors’ interests (Panjwani and Caraher, 2014).

Another thoroughly researched and discussed topic is individuals’ personal responsibility for their health (Wikler, 2002; Nihlén Fahlquist, 2019). While the focus of public health is on promoting the health of the general population, research has shown that actions focusing on promoting the general population’s health may not benefit every individual (Nihlén Fahlquist, 2019) and that it is also important to focus on different subgroups. Therefore, the results of this study showing that politicians rate societal responsibility lower for newly arrived migrants’ health indicate a potential problem. Migrants are a group with an increased risk of ill health (Rechel *et al.*, 2013; Norredam *et al.*, 2014; Helgesson *et al.*, 2019). Another interesting aspect is that although politicians rate societal responsibility lower, they also rate individual responsibility for newly arrived migrants lower. They also rate newly arrived migrants’ possibility lower than their responsibility to promote health. This is in line with previous research showing that migrants are often not reached to the same degree as the general population, for example, through preventive actions, such as cancer screening (Lagerlund *et al.*, 2002). Previous research has shown that high ratings of societal and individual responsibility for health are not mutually exclusive (Traina *et al.*, 2019), which is in line with the findings in the present study.

The high ratings of the state’s responsibility and possibility to promote health in this study are interesting. In Sweden, it is the regions that are responsible for healthcare organization and delivery (Swedish Association of Local Authorities and Regions, 2021b), which explains their high ratings in this study. The state has no such responsibility for healthcare. However, it does have the ability to offer regions extra financial support for specific projects, as it has done previously (Government Office of Sweden, 2016). A previous study also highlighted the importance of the role of states in health promotion, for example by providing and protecting human rights and services, establishing policy frameworks that facilitate equitable health improvements, and gathering and monitoring health-related data about the population. The same study also showed how important it is for governments to protect minorities’ rights and opportunities for health (Blas *et al.*, 2008).

Politicians rated civil society’s responsibility and possibility to promote newly arrived migrants’ health lower than those of other actors. However, previous research has demonstrated the important role of civil society as a ‘driving force’ in health promotion, as it can influence governmental actors on the local, national and global levels (Blas *et al.*, 2008).

The analysis of subgroups and patterns in this study points to two main groups of politicians: those who rate responsibility and possibility to promote newly arrived migrants’ health high and those who rate them low. This indicates that the main difference between politicians lies in whether they see society as a whole, rather than specific actors, as having the responsibility and possibility to promote health. As previous research in this area is scarce, these results are interesting. However, further research is needed to improve the understanding of this phenomenon and its implications.

The factors that were found to be important for politicians’ high ratings of societal responsibility and possibility in this study are generally in line with research into the factors that affect politicians in their work. For example, gender has been shown to affect the areas in which politicians are active (Campbell and Winters, 2008) and to be an important factor when politicians reflect on their considerations of newly arrived migrants’ health in their decision-making (Svanholm *et al.*, 2021).

A previous study found that regional politicians rated their influence on decision-making in health as weaker than that of other involved actors, such as physicians and administrators (Rosén *et al.*, 2014). In this study, regional politicians’ responsibility and possibility were rated higher than those of other actors. However, the results also indicate that municipality politicians are more likely to rate societal responsibility and possibility to promote health high. This is positive, since it is important for other actors outside the healthcare sector to contribute to health promotion (De Leeuw, 2017). Another important factor for politicians’ high ratings of societal responsibility and possibility to promote health was personal interest in public health. This is in line with previous research that has identified personal interest as important for politicians’ active involvement in health promotion (Zalmanovitch and Cohen, 2015). Lastly, attitude—believing that the effects of decision-making on newly arrived migrants’ health are important—is an important factor for politicians’ high ratings of societal responsibility. Similarly, attitude has been shown to be important for whether politicians consider health effects on newly arrived migrants’ health in their decision-making (Svanholm *et al.*, 2021). Thus, our findings support previous research showing that attitude and knowledge are closely associated with practice in health (World Health Organization, 2012). As personal characteristics are important for whether politicians rate societal responsibility and possibility high or low, this could indicate insufficient structural factors supporting politicians in their health promotion roles.

The results of the current study are important both from a practical and theoretical standpoint. The results could be utilized in an evaluation of current structural factors and practices of how politicians work and how they promote health, to improve these. It is also important when forming educational or lobbying projects aimed at affecting politicians’ attitudes or actions regarding promoting health. Further studies are needed to deepen the understanding of politicians’ role in health promotion. While follow-up studies could identify change over time, qualitative studies could offer a deeper understanding of how politicians view the difference between the whole population and migrants more specifically, or why women tend to rate politicians’ responsibility and possibility to promote health as higher than male politicians do.

### Limitations

Although this study has several strengths, it is important to highlight certain limitations. This research project was the first to use the questionnaire, whose development was described in detail previously (Svanholm *et al.*, 2021).

Cluster analysis is described as an exploratory technique (Hair *et al.*, 2010) and was therefore a good fit for this study. There is no one way but several suggested ways of assessing validity in cluster analysis (Henry *et al.*, 2005; Hair *et al.*, 2010). In this study, validity was assessed by performing a separate cluster analysis with randomly selected participants and then repeating the analysis with the rest of the participants to ensure that the solutions did not differ significantly (Hair *et al.*, 2010). Hierarchical and nonhierarchical cluster analyses were performed to take advantage of the strengths of both (Henry *et al.*, 2005; Hair *et al.*, 2010).

The methodological literature emphasizes that clusters are generated by specific data and are therefore difficult to generalize to other populations (Henry *et al.*, 2005; Hair *et al.*, 2010). This, as well as the fact that the questionnaire is new, warrants caution when generalizing the results of this study. However, many of the findings are in line with previous research, thus adding to the relevant knowledge.

## CONCLUSION

Municipality and regional politicians in northern Sweden rate both societal and individual responsibility and possibility to promote health high. Generally, they rate responsibility and possibility to promote health higher with regard to the general population than with regard to newly arrived migrants. The state and regions are viewed as the actors with the greatest responsibility and possibility, while civil society and private actors are seen as having the least. Politicians can be divided into those who rate responsibility and possibility to promote newly arrived migrants’ health high and those who rate them low. Factors important for politicians’ high ratings of societal responsibility and possibility are believing that it is important to consider the health effects of decision-making on newly arrived migrants, rating one’s knowledge of newly arrived migrants’ health status as good, being a municipality politician, having a personal interest in public health, and being female.

## ETHICS

Ethics approval for the study was obtained from the Research Ethics Committee of Mid Sweden University (MIUN 2019/6).
